# Supplementary material for: An IL-2-grafted antibody immunotherapy with potent efficacy against metastatic cancer
Source: Nat Commun. 2020 Dec 22;11:6440. doi: 10.1038/s41467-020-20220-1 (PMC7755894; doi:10.1038/s41467-020-20220-1)
Supplement: Supplementary file 1 — Supplementary Information [file 41467_2020_20220_MOESM1_ESM.pdf]

Supplementary Information

An IL-2-grafted antibody immunotherapy with potent efficacy against metastatic cancer

Dilara Sahin[1], Natalia Arenas-Ramirez[1], Matthias Rath[1], Ufuk Karakus[1], Monika Hümbelin[1], Merel van Gogh[2], Lubor Borsig[2], and Onur Boyman[1,3]*

[1] Department of Immunology, University Hospital Zurich, CH-8091 Zurich, Switzerland

[2] Institute of Physiology, University of Zurich, CH-8057 Zurich, Switzerland

[3] Faculty of Medicine, University of Zurich, CH-8006 Zurich, Switzerland

* Corresponding author: onur.boyman@uzh.ch

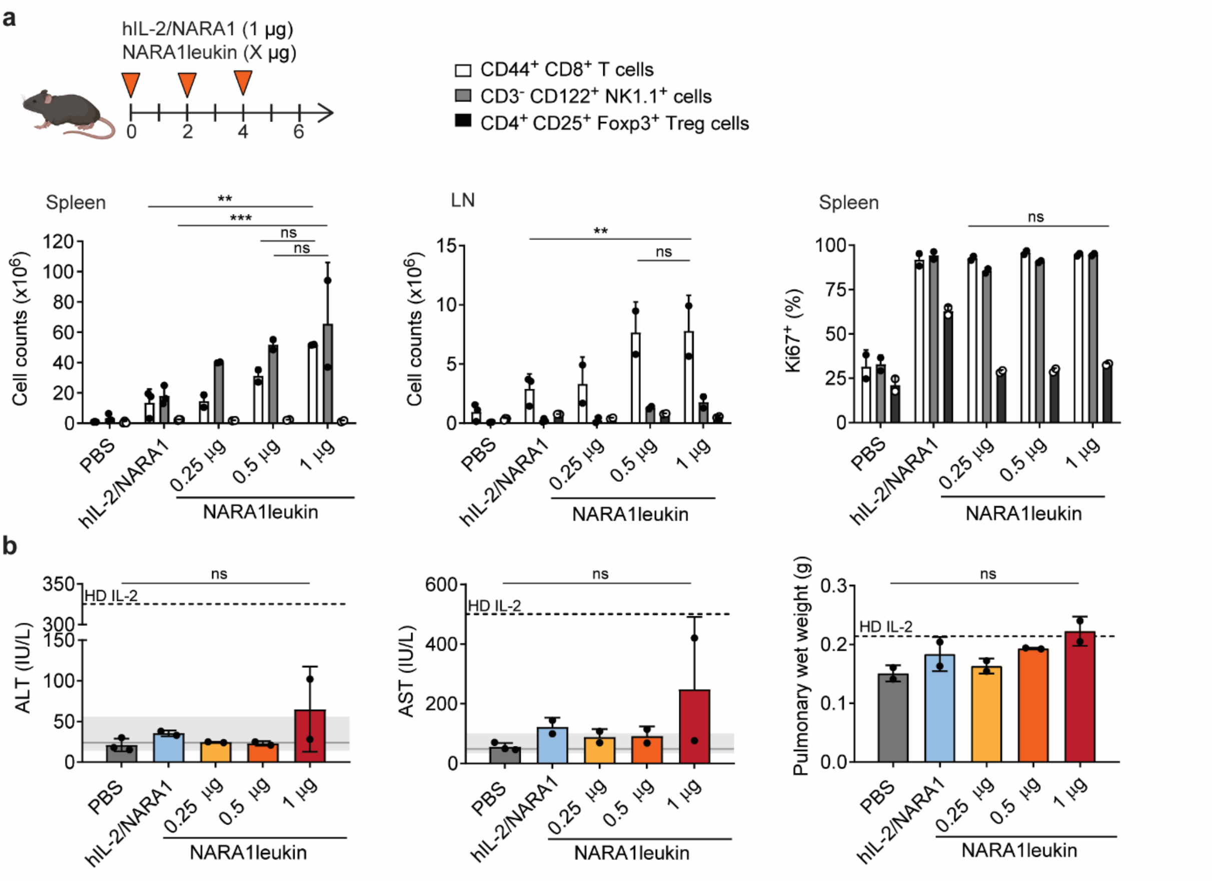

Supplementary Figure 1. Dose escalation and toxicity of NARA1leukin.

(**a**, **b**) Mice received three injections of PBS, hIL-2/NARA1 complexes (1 µg / 5 µg), or titrated doses of NARA1leukin (0.25, 0.5 or 1 µg hIL-2 equivalent). (**a**) Spleens and lymph nodes (LN) were analyzed on day 6 to determine total cells counts and Ki67 abundance of $CD44^+$ $CD8^+$ T cells, $CD3^-$ $CD122^+$ $NK1.1^+$ NK cells, and $CD4^+$ $CD25^+$ $Foxp3^+$ regulatory T (Treg) cells. ***p* = 0.0025 (Spleen), ****p* = 0.0002, ***p* = 0.0008 (LN). (**b**) Concentrations of aspartate aminotransferase (AST) and alanine aminotransferase (ALT) were measured in sera collected on day 6. Lungs were harvested to measure pulmonary wet weight (in gram, g). Dashed lines indicate the levels obtained with high-dose hIL-2 at 100 µg injected for 7 consecutive days[1], gray areas indicate the physiologic ranges of AST and ALT concentrations in the serum, and the gray lines show the mean[2]. Data are presented as mean ± SD of two independent experiments, with n = 3 (PBS and hIL-2/NARA1, counts), n = 2 (NARA1leukin, counts and all groups, Ki67) (**a**) n = 3 (PBS, hIL-2/NARA1, AST and ALT), n = 2 (NARA1leukin, AST and ALT and all groups, pulmonary wet weight) mice/group. Differences between groups at the same time point were analyzed using two-way ANOVA followed by Tukey's multiple comparison test (**a**) or Kruskal-Wallis test followed by Dunn's multiple comparison test (**b**). ns, not significant. Source data are provided as a Source Data file.

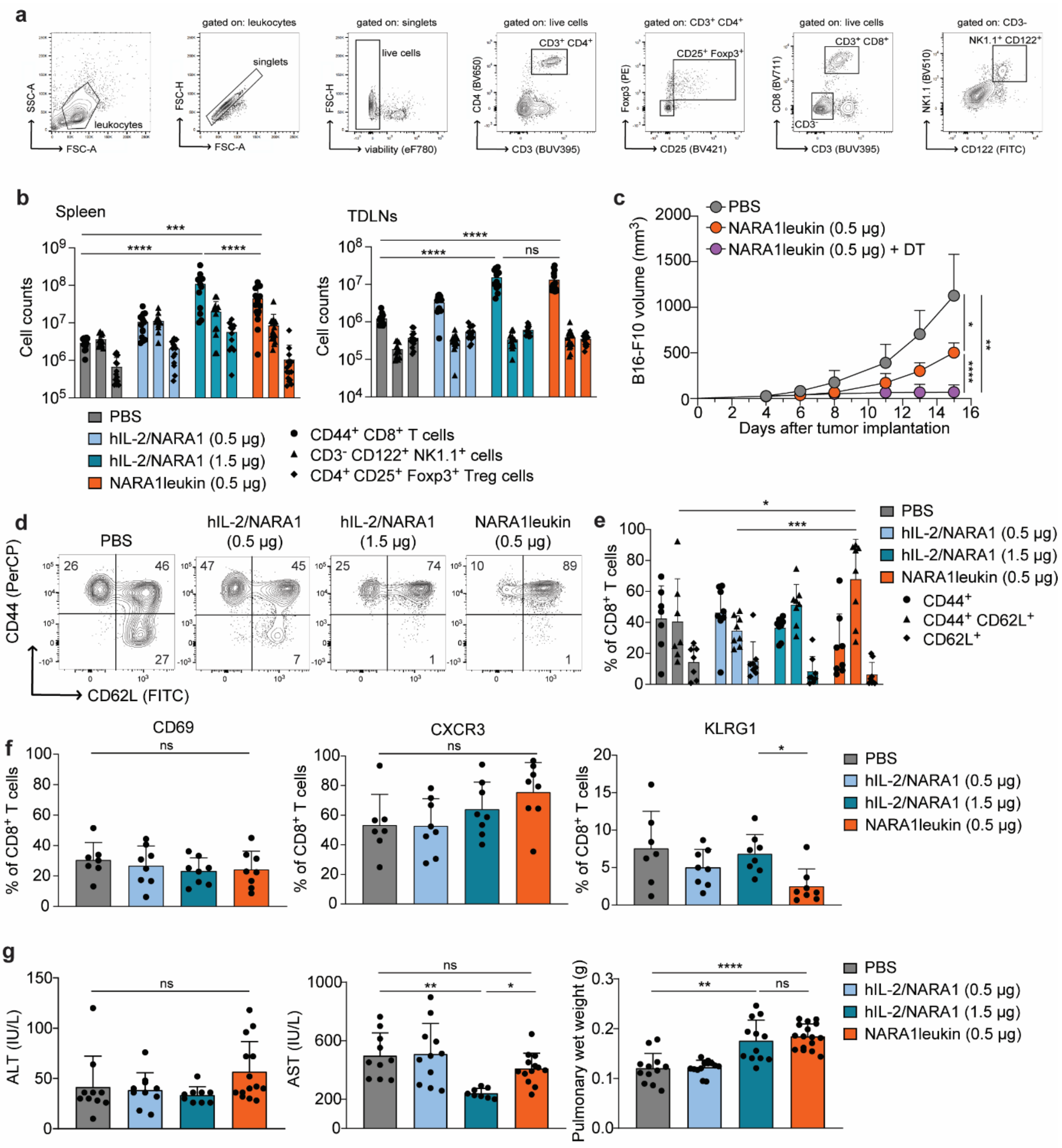

Supplementary Figure 2. Anti-tumor immune responses and toxicity of NARA1leukin.

(**a**) Single cell suspension obtained from spleen of a B16-F10-bearing, PBS-treated mouse was used as an example to demonstrate our gating strategy for indicated immune cell subsets. (**b**) Mice were injected intradermally with B16-F10 melanoma cells on day 0 followed by injections of PBS, hIL-2/NARA1 complexes, or NARA1leukin, as in **Fig. 4a**. On day 15, spleens and tumor-draining lymph nodes (TDLNs) were analyzed for cell counts of indicated immune cell subsets. ***p = 0.001, ****p < 0.0001. (**c**) *Foxp3^{DTR}* mice were injected intradermally with B16-F10 melanoma cells on day 0 and treated with PBS, NARA1leukin (as in **Fig. 4a**), or NARA1leukin and diphtheria toxin (DT, 0.5 µg, twice weekly) from day 4 to 14. Shown are

tumor growth curves of intradermal B16-F10 nodules. *p = 0.024, **p = 0.0018, ****p < 0.0001. (**d**-**g**) Mice were injected intradermally with B16-F10 melanoma cells on day 0 followed by injections of PBS, hIL-2/NARA1 complexes, or NARA1leukin (as in **Fig. 4a**). On day 15, tumor infiltrating $CD8^+$ T cells were analyzed by flow cytometry. Shown are representative flow cytometry dot plots (**d**) and percentages (**e**, **f**) of $CD8^+$ T cells. *p = 0.0109, ***p = 0.0008, *p = 0.03 (**g**) Sera were collected from intradermal B16-F10-bearing animals to determine concentrations of AST and ALT, and lungs were harvested to measure pulmonary wet weight. **p = 0.01 (AST), *p = 0.02, ****p < 0.0001, **p = 0.002. Data are presented as mean ± SD of four (**b**, **g**), two (**c**) or three (**e, f**) independent experiments, with n = 13 (PBS), n = 14 (hIL-2/NARA1 0.5 μg), n = 12 (hIL-2/NARA1 1.5 μg), n = 16 (NARA1leukin) (**b**), n = 7 (PBS and DT), n = 8 (NARA1leukin) (**c**), n = 7 (PBS), n = 8 (hIL-2/NARA1 0.5 μg/1.5 μg and NARA1leukin) (**e**, **f**), n = 10 (PBS, hIL-2/NARA1 0.5 μg), n = 9 (hIL-2/NARA1 1.5 μg), n = 14 (NARA1leukin) (**g**, ALT), n = 10 (PBS), n = 11 (IL-2/NARA1 0.5 μg), n = 8 (hIL-2/NARA1 1.5 μg), n = 13 (NARA1leukin) (**g**, AST), n = 13 (PBS), n = 14 (hIL-2/NARA1 0.5 μg), n = 12 (hIL-2/NARA1 1.5 μg), n = 16 (NARA1leukin) (**g**, pulmonary wet weight) mice/group. Differences between groups at the same time point were analyzed using two-way ANOVA followed by Tukey's multiple comparison test (**b**, **c**, **e**) or Kruskal-Wallis test followed by Dunn's multiple comparison test (**f**, **g**). ns, not significant. Source data are provided as a Source Data file.

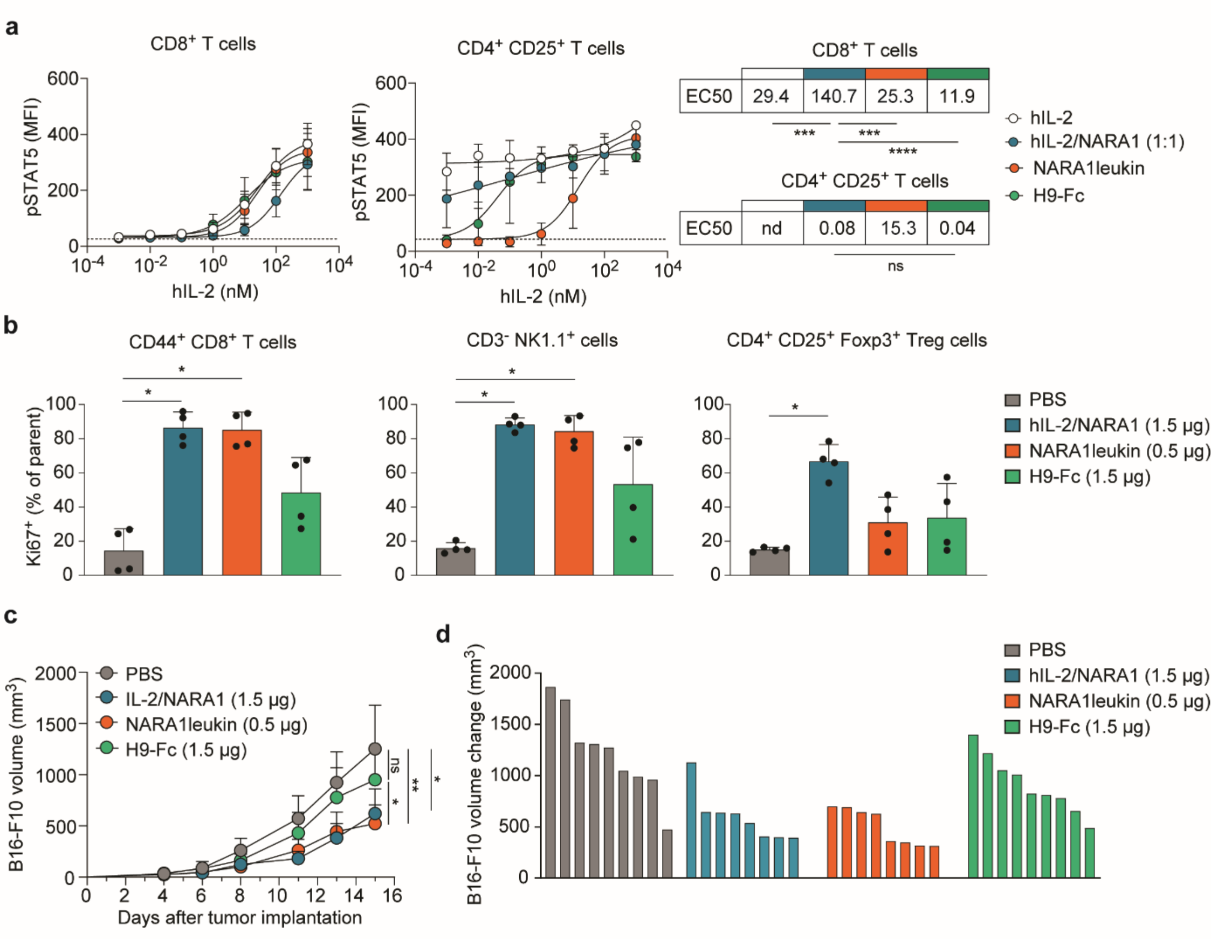

Supplementary Figure 3. ***In vitro*** **and** ***in vivo*** **evaluation of NARA1leukin in comparison to H9-Fc.**

(**a**) Phosphorylated STAT5 (pSTAT5) levels of mouse immune cell subsets responding to titrated hIL-2, hIL-2/NARA1, NARA1leukin, or H9-Fc. Dotted lines indicate mean fluorescent intensity (MFI) of pSTAT5 of indicated cell subsets following incubation in media for 15 minutes. Differences between curves were analyzed using one-way ANOVA followed by Tukey's multiple comparison test. nd: not determined. ***p = 0.0005 (hIL-2), ***p = 0.0001 (hIL-2/NARA1), ****p < 0.0001 (**b**) Mice received three injections of PBS, hIL-2/NARA1 complexes (1.5 µg / 15 µg), NARA1leukin (0.5 µg hIL-2 equivalent), or H9-Fc (1.5 µg hIL-2 equivalent). Spleens were analyzed on day 6 to determine Ki67 abundance in CD44$^+$ CD8$^+$ T cells, CD3$^-$ CD122$^+$ NK1.1$^+$ NK cells, and CD4$^+$ CD25$^+$ Foxp3$^+$ Treg cells. CD8: *p = 0.0227 (NARA1leukin), *p = 0.014 (hIL-2/NARA1); NK: *p = 0.036 (NARA1leukin), *p = 0.014 (hIL-2/NARA1); Treg: *p = 0.0109. (**c**, **d**) Mice were injected intradermally with B16-F10 melanoma cells and treated with PBS, hIL-2/NARA1 complexes (1.5 µg /15 µg, three times weekly), NARA1leukin (0.5 µg hIL-2 equivalent, twice weekly), or H9-Fc (1.5 µg hIL-2 equivalent, three times weekly) from day 4 to 14. Shown are tumor growth curves of intradermal nodules (**c**) and change in tumor volume between day 4 and 15 (**d**). *p = 0.0102

(PBS), **p = 0.0031, *p = 0.0121 (H9-Fc). Data are presented as mean ± SD of two independent experiments, with n = 4 (**b**) or n = 9 (PBS and H9-Fc), n = 8 (hIL-2/NARA1 and NARA1leukin) (**c**, **d**) mice/group. Differences between groups were analyzed using Kruskal-Wallis test followed by Dunn's multiple comparison test (**b**) or two-way ANOVA followed by Tukey's multiple comparison test (**c**). ns, not significant. Source data are provided as a Source Data file.

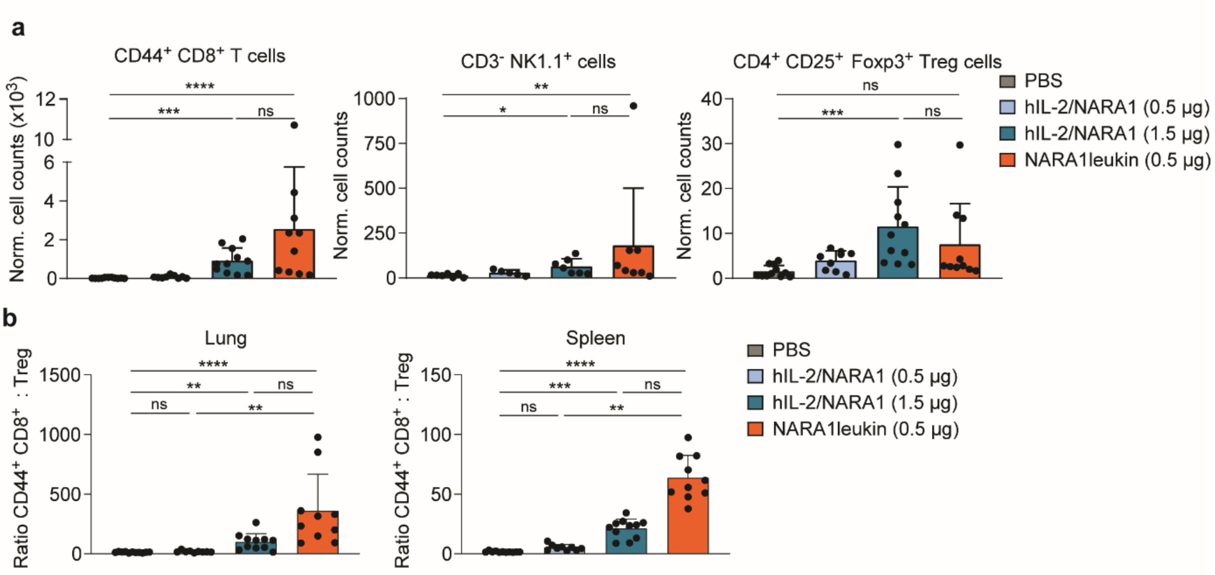

Supplementary Figure 4. Anti-tumor immune responses by NARA1leukin in pulmonary B16-F10 lesions.

(**a, b**). B16-F10 pulmonary nodule-bearing animals were treated with PBS, hIL-2/NARA1 complexes, or NARA1leukin (as in **Fig. 5a**). Spleens and lungs were analyzed on day 18 by flow cytometry for the indicated immune cell subsets. (**a**) Cell counts of $CD44^+$ $CD8^+$ T cells, $CD3^-$ $CD122^+$ $NK1.1^+$ NK cells, and $CD4^+$ $CD25^+$ $Foxp3^+$ Treg cells in lungs are shown, normalized to the count of pulmonary nodules. ****$p < 0.0001$, ***$p = 0.0001$ (CD8), **$p = 0.0093$, *$p = 0.0383$, ***$p = 0.0002$ (Treg). (**b**) Ratios of $CD44^+$ $CD8^+$ T to $CD4^+$ $CD25^+$ $Foxp3^+$ Treg cells for lungs and spleens are shown. ****$p < 0.0001$, **$p = 0.0027$ (lung, PBS), **$p = 0.0011$ (lung, hIL-2/NARA1), ***$p = 0.0007$, **$p = 0.0013$ (spleen). Data are presented as mean ± SD of three independent experiments (two for NK cells), with $n = 11$ (PBS, hIL-2/NARA1 1.5 µg), $n = 9$ (hIL-2/NARA1 0.5 µg), $n = 10$ (NARA1leukin) (**a**, T cells, **b**), $n = 7$ (PBS, hIL-2/NARA1 1.5 µg), $n = 5$ (hIL-2/NARA1 0.5 µg), $n = 8$ (NARA1leukin) (**a**, NK cells) mice/group. Differences between groups were analyzed using Kruskal-Wallis test followed by Dunn's multiple comparison test. ns, not significant. Source data are provided as a Source Data file.

Supplementary table 1: Antibodies used for flow cytometry.

Antigen	Fluorophore	Clone	Dilution	Company	Serial number
mCD122	PE	5H4	1:200	eBioscience	12-1221
mCD122	FITC	TM-β1	1:200	BD Biosciences	553361
mCD183	BV421	CXCR3-173	1:100	BioLegend	126529
mCD25	APC	PC61	1:200	BD Biosciences	557192
mCD25	BV421	PC61	1:200	BioLegend	102034
mCD3	BV510	145-2C11	1:100	BD Biosciences	563024
mCD3	FITC	145-2C11	1:400	eBioscience	11-0031
mCD3	BV395	145-2C11	1:100	BD Biosciences	563565
mCD4	BV605	GK1.5	1:400	BioLegend	100451
mCD4	BV650	RM4-5	1:400	BioLegend	100545
mCD44	APC	IM7	1:200	eBioscience	17-0441
mCD44	PerCP	IM7	1:200	BioLegend	103036
mCD45.2	Alexa-700	104	1:400	BioLegend	109822
mCD45.2	BUV395	104	1:200	BD Biosciences	564616
mCD45.2	BUV737	104	1:200	BD Biosciences	564880
mCD62L	FITC	MEL-14	1:100	Invitrogen	11-0621-82
mCD69	BV711	H1.2F3	1:100	BioLegend	104537
mCD8a	Alexa 488	53-6.7	1:400	eBioscience	53-0081
mCD8a	BV711	53-6.7	1:400	BioLegend	100747
mCD8b	APC-efluor 780	H35-17.2	1:400	eBioscience	47-0083
mFoxp3	PE	FJK-16s	1:200	eBioscience	12-5773
Ki-67	Alexa Fluor647	B56	1:200	BD Biosciences	561126
KLRG	PE-Cy7	2F1/KLRG1	1:100	BioLegend	138416
mNK1.1	BUV395	PK136	1:100	BD Biosciences	564144
mNK1.1	BV510	PK136	1:100	BD Biosciences	563096
mPD-1	BV605	29F.1A12	1:100	BioLegend	135219
STAT5(pY694)	Alexa Fluor647	47/Stat5(pY694)	1:25	BD Biosciences	562076
STAT5(pY694)	FITC	Polyclonal	1:25	eBiosciences	11-9010
mTIM-3	PE-Cy7	RMT3-23	1:100	eBiosciences	25-5870
TOX	eF660	TXRX10	1:100	Invitrogen	50-6502-80
hCD3	BV510	OKT3	1:200	BioLegend	317332
hCD4	BV650	RPA-T4	1:200	BioLegend	300536
hCD25	APC	M-A251	1:100	BioLegend	356109
hCD56	BV421	HCD56	1:200	BioLegend	318328

Supplementary References

1. Arenas-Ramirez N, *et al.* Improved cancer immunotherapy by a CD25-mimobody conferring selectivity to human interleukin-2. *Sci Transl Med* **8**, 367ra166 (2016).
2. Otto GP, *et al*. Clinical Chemistry Reference Intervals for C57BL/6J, C57BL/6N, and C3HeB/FeJ Mice (Mus musculus). *J Am Assoc Lab Anim Sci* **55(4)**, 375-386. (2016)
