# Supplementary material for: An IL-2-grafted antibody immunotherapy with potent efficacy against metastatic cancer
Source: Nat Commun. 2020 Dec 22;11:6440. doi: 10.1038/s41467-020-20220-1 (PMC7755894; doi:10.1038/s41467-020-20220-1)
Supplement: Supplementary file 3 — Reporting Summary [file 41467_2020_20220_MOESM3_ESM.pdf]

nature research

Corresponding author(s): Onur Boyman

Last updated by author(s): Nov 4, 2020

Reporting Summary

Nature Research wishes to improve the reproducibility of the work that we publish. This form provides structure for consistency and transparency in reporting. For further information on Nature Research policies, see Authors & Referees and the Editorial Policy Checklist .

Statistics

For all statistical analyses, confirm that the following items are present in the figure legend, table legend, main text, or Methods section.

n/a	Confirmed	
☐	☒	The exact sample size (*n*) for each experimental group/condition, given as a discrete number and unit of measurement
☐	☒	A statement on whether measurements were taken from distinct samples or whether the same sample was measured repeatedly
☐	☒	The statistical test(s) used AND whether they are one- or two-sided<br>*Only common tests should be described solely by name; describe more complex techniques in the Methods section.*
☒	☐	A description of all covariates tested
☐	☒	A description of any assumptions or corrections, such as tests of normality and adjustment for multiple comparisons
☐	☒	A full description of the statistical parameters including central tendency (e.g. means) or other basic estimates (e.g. regression coefficient) AND variation (e.g. standard deviation) or associated estimates of uncertainty (e.g. confidence intervals)
☐	☒	For null hypothesis testing, the test statistic (e.g. *F*, *t*, *r*) with confidence intervals, effect sizes, degrees of freedom and *P* value noted<br>*Give P values as exact values whenever suitable.*
☒	☐	For Bayesian analysis, information on the choice of priors and Markov chain Monte Carlo settings
☒	☐	For hierarchical and complex designs, identification of the appropriate level for tests and full reporting of outcomes
☒	☐	Estimates of effect sizes (e.g. Cohen's *d*, Pearson's *r*), indicating how they were calculated

Our web collection on statistics for biologists contains articles on many of the points above.

Software and code

Policy information about availability of computer code

Data collection	Data was collected with BD FACSDiva™ 8.0.1. Software.
Data analysis	Data was analysed with Graphpad Prism 8, Microsoft Excel 16, and BD FlowJo 10.

For manuscripts utilizing custom algorithms or software that are central to the research but not yet described in published literature, software must be made available to editors/reviewers. We strongly encourage code deposition in a community repository (e.g. GitHub). See the Nature Research guidelines for submitting code & software for further information.

Data

Policy information about availability of data

All manuscripts must include a data availability statement. This statement should provide the following information, where applicable:

- Accession codes, unique identifiers, or web links for publicly available datasets
- A list of figures that have associated raw data
- A description of any restrictions on data availability

No big data set was used in the study. The data supporting the findings of this study are available within the paper and its supplementary information files. Source data are provided with this paper that contains raw data presented in Figs. 2–6 and in Supplementary Figs. 1–4.

Field-specific reporting

Please select the one below that is the best fit for your research. If you are not sure, read the appropriate sections before making your selection.

☒ Life sciences ☐ Behavioural & social sciences ☐ Ecological, evolutionary & environmental sciences

October 2018

Life sciences study design

All studies must disclose on these points even when the disclosure is negative.

Sample size	Sample size was calculated with R studio using data from pilot or previous experiments with interleukin-2 complexes (Arenas-Ramirez, 2016, Sci Transl. Med.). As measured parameters are continuous values, power.anova.test was used with an alpha error of 5% (0.05) and a beta error of 20% (Power = 80%). For certain experimental setups with high variability and/or modest treatment effects, sample size was calculated to be higher.
Data exclusions	In Fig. 6h-i, one mouse in the hIL-2/NARA1 group was excluded as it reached euthanasia criteria before the end of experiment. In Supp. Fig. 4 a,b one mice in hIL-2/NARA1 (0.5 ug) group was excluded as the lung cells were lost during processing and no events were detectable in flow cytometry. In Supp. Fig. 2, ALT and AST values were not included from hemolytic blood samples.
Replication	All experiments were repeated independently and on different days at least two times. Replications showed the same trend and pooled data are displayed throughout the manuscript. Three replication experiments of the intravenous B16-F10 (pulmonary) model (two repetitions of Fig. 5a-d and one repetition of Fig. 5g-j) were excluded because positive and negative controls did not work. The number of replicated experiments are defined within the figure legends.
Randomization	Mice were assigned to the respective treatment groups in a randomized fashion. For in vitro experiments using human or mouse cells same sample was equally distributed to all groups.
Blinding	Tumor measurements, metastatic nodule counting and survival assessment were done by a blinded technician or researcher. Other analyses performed by using flow cytometry or ELISA was done unblinded as the data collection was quantitative and unbiased by nature.

Reporting for specific materials, systems and methods

We require information from authors about some types of materials, experimental systems and methods used in many studies. Here, indicate whether each material, system or method listed is relevant to your study. If you are not sure if a list item applies to your research, read the appropriate section before selecting a response.

Materials & experimental systems

n/a	Involved in the study	
☐	☒	Antibodies
☐	☒	Eukaryotic cell lines
☒	☐	Palaeontology
☐	☒	Animals and other organisms
☐	☒	Human research participants
☒	☐	Clinical data

Methods

n/a	Involved in the study	
☒	☐	ChIP-seq
☐	☒	Flow cytometry
☒	☐	MRI-based neuroimaging

Antibodies

Antibodies used	Information on all antibodies used in this study are provided in Supplementary Table 1.
Validation	Antibodies were validated by respective manufacturers for flow cytometry by staining of human or mouse primary cells or cell lines. Validation statements as well as references from the literature can be found on the manufacturers' websites. The reactivity of the antibodies are indicated with "m" for mouse and "h" for human. Antibodies that do not have an indication are reported to be cross-reactive for mouse and human e.g. pSTAT5, TOX, Ki67. Antibodies were not validated by/for other techniques than flow cytometry.

Eukaryotic cell lines

Policy information about cell lines

Cell line source(s)	293T (ATCC® CRL-3216™), B16-F10 (ATCC® CRL-6475™), LLC1 (ATCC® CRL-1642™) and 4T1 (ATCC® CRL-2539™) cell lines were obtained from ATCC. FreeStyle™ CHO-S Cells were obtained from Thermo Fisher Scientific.
Authentication	Only cells derived from the original aliquots were used within the study. All certificates can be obtained from the official ATCC and Thermo Fisher website. https://www.lgcstandards-atcc.org/products/all/crl-3216.aspx?geo_country=ch, https://www.lgcstandards-atcc.org/products/all/CRL-6475.aspx?geo_country=ch#documentation, https://www.lgcstandards-atcc.org/products/all/CRL-1642.aspx?geo_country=ch#documentation, https://www.lgcstandards-atcc.org/Products/All/CRL-2539.aspx?geo_country=ch#documentation, https://www.thermofisher.com/order/catalog/product/R80007#/R80007

Mycoplasma contamination

The initial vial of cells were expanded to establish a stock, which was tested negative for mycoplasma. Only cells from this stock were used in this study.

Commonly misidentified lines (See ICLAC register)

No such cell lines were used in this study.

Animals and other organisms

Policy information about studies involving animals; ARRIVE guidelines recommended for reporting animal research

Laboratory animals

C57Bl/6J mice were purchased from Charles River Laboratories. Balb/c (JAX Stock No:006584) mice were obtained from the Jackson Laboratory and bred in house. Female mice were used for experiments at 2-3 months of age.

Wild animals

No wild animals were used in this study.

Field-collected samples

This study did not include field-collected samples.

Ethics oversight

Animal experiments received prior approval by the veterinary office of the Canton of Zurich (License numbers 142/2017 and 246/2016) and were conducted in accordance with Swiss Federal and Cantonal Law.

Note that full information on the approval of the study protocol must also be provided in the manuscript.

Human research participants

Policy information about studies involving human research participants

Population characteristics

Anonymized buffy coats were obtained from the Swiss Blood Bank Zurich. Characteristic data on the donors is not available

Recruitment

Anonymized buffy coats were obtained from the Swiss Blood Bank Zurich.

Ethics oversight

The “Fundamental research project for phenotypical and functional characterization of different leukocyte subsets in healthy and diseased individuals” (PFCL-1, BASEC no. 2016-01440) project has been reviewed and approved by the Kantonale Ethikkommission Zurich and has been carried out in accordance with principles enunciated in the current version of the Declaration of Helsinki, the guidelines of Good Clinical Practice, and Swiss legal requirements.

Note that full information on the approval of the study protocol must also be provided in the manuscript.

Flow Cytometry

Plots

Confirm that:

- [x] The axis labels state the marker and fluorochrome used (e.g. CD4-FITC).
- [x] The axis scales are clearly visible. Include numbers along axes only for bottom left plot of group (a 'group' is an analysis of identical markers).
- [x] All plots are contour plots with outliers or pseudocolor plots.
- [x] A numerical value for number of cells or percentage (with statistics) is provided.

Methodology

Sample preparation

Single cell suspensions of lymph nodes and spleens were prepared according to standard protocols. Tumors were cut into small pieces, and incubated in 10 ml dissociation buffer (RPMI, 5% FCS, 10 ug/ml DNAse I (Sigma-Aldrich), and 200 U/ml collagenase type I (Thermo Fisher Scientific) for 60 min at 37°C and shaking with 25 rpm. Cell suspensions were then passed through a 70 µm cell strainer. In experiments with intradermally implanted tumors, after one wash a Percoll (40% and 70%; GE Healthcare) gradient centrifugation was performed. Lungs were passed through a 70 µm cell strainer followed by Percoll gradient centrifugation. All cell suspensions were stained for flow cytometry analysis using flow cytometry buffer (PBS with 2% FCS, 2 mM EDTA) and fluorochrome-conjugated antibodies for at least 20 min at 4°C. A list of used antibodies is provided in Supplementary Table 1. Intracellular staining with Ki67 and Foxp3 was performed following manufacturer’s instructions (eBioscience™ Foxp3 / Transcription Factor Staining Buffer Set, ThermoFisher).
For STAT5 phosphorylation analysis, freshly isolated mouse splenocytes or human PBMCs were incubated for 15 min at 37°C with indicated formulations. Cells were directly fixed with Fix Buffer I (BD) for 10 min at 37°C followed by permeabilization with Perm Buffer III (BD Phosflow™) and staining with fluorochrome-conjugated antibodies in flow cytometry buffer. For in vivo pSTAT5 spleens were directly fixed in Lyse/Fix Buffer (BD Phosflow™) followed by permeabilization with Perm Buffer III (BD Phosflow) for 1h and staining with fluorochrome-conjugated antibodies .

Instrument

Samples were acquired with a BD LSR II flow cytometer (BD Biosciences).

Software

Data was collected and analysed by BD FACS DIVA and BD FlowJo 10.

Cell population abundance

Cell sorting was not used in this study.

Gating strategy

The markers used to define immune cell populations are provided in the figures. Mouse cells: FFC/SSC gate was set to include all leukocytes but exclude cell debris. FFC-H/FFC-A gate was used to gate on singlets. Dead cells were excluded by Fixable Viability Dye eFluor™ 780 (eBioscience™). Following populations were gated on live cells: CD3+CD4+ (CD4 T cells), CD3+CD8+ (CD8 T cells), CD3+CD4+CD25+Foxp3+ (T regulatory cells) and CD3-NK1.1+CD122+ (NK cells). CD44high CD8 T cells were gated on CD8 T cells. Ki67, pSTAT5, PD-1 and TIM-3 positive fractions were gated based on FMO staining. Human cells: FFC/SSC gate was set to include all leukocytes but exclude cell debris. FFC-H/FFC-A gate was used to gate on singlets. Following populations were gated on single cells: CD3+CD4+ (CD4 T cells), CD3+CD4+CD25+ (CD25+ CD4 T cells), CD3+CD4- (cytotoxic T cells) and CD3-CD56+ (NK cells).

☒ Tick this box to confirm that a figure exemplifying the gating strategy is provided in the Supplementary Information.
